# Chimeric flavivirus enables evaluation of antibodies against dengue virus envelope protein in vitro and in vivo

**DOI:** 10.1038/s41598-020-78639-x

**Published:** 2020-12-09

**Authors:** Takeshi Kurosu, Keiko Hanabara, Azusa Asai, Sabar Pambudi, Supranee Phanthanawiboon, Magot Diata Omokoko, Ken-ichiro Ono, Masayuki Saijo, Pongrama Ramasoota, Kazuyoshi Ikuta

**Affiliations:** 1 Research Institute for Microbial Diseases (RIMD), Osaka University, Suita, Osaka 565-0871 Japan; 2 Medical and Biological Laboratories CO., LTD., Ina, Nagano 396-0002 Japan; 3 Department of Virology I, National Institute of Infectious Diseases, 4-7-1 Gakuen, Musashimurayama-shi, Tokyo, 208-0011 Japan; 4 Center of Excellence of Antibody Research, Department of Social and Environmental Medicine, Faculty of Tropical Medicine, Mahidol University, Bangkok, 10400 Thailand; 5 Present Address: Department of Virology I, National Institute of Infectious Diseases, 4-7-1 Gakuen, Musashimurayama-shi, Tokyo, 208-0011 Japan

**Keywords:** Microbiology, Virology, Dengue virus

## Abstract

In a secondary dengue virus (DENV) infection, the presence of non-neutralizing antibodies (Abs), developed during a previous infection with a different DENV serotype, is thought to worsen clinical outcomes by enhancing viral production. This phenomenon is called antibody-dependent enhancement (ADE) of infection, and it has delayed the development of therapeutic Abs and vaccines against DENV, as they must be evaluated for the potential to induce ADE. Unfortunately, limited replication of DENV clinical isolates in vitro and in experimental animals hinders this evaluation process. We have, therefore, constructed a recombinant chimeric flavivirus (DV2ChimV), which carries premembrane (prM) and envelope (E) genes of type 2 DENV (DENV-2) R05-624 clinical (Thai) isolate in a backbone of Japanese encephalitis virus (Nakayama strain). DENV E-protein is the most important viral target, not only for neutralizing Abs, but also for infection-enhancing Abs. In contrast to DENV-2 R05-624, DV2ChimV replicated efficiently in cultured mammalian cells and was lethal in interferon-α/β–γ-receptor double-knockout mice. With DV2ChimV, we were able to perform neutralization assays, in vitro and in vivo ADE assays, and in vivo protection assays. These results suggest that the chimeric virus is a powerful tool for evaluation of Abs against DENV.

## Introduction

Dengue fever, an arthropod-borne disease caused by dengue virus (DENV, of the family *Flaviviridae* and the genus *Flavivirus*), is a major public-health problem in tropical and subtropical regions^1^. The DENV genome encodes the capsid, premembrane (prM), and envelope (E) structural proteins, and the NS1, NS2a, NS2b, NS3, NS4a, NS4b, and NS5 nonstructural proteins^2^. Primary DENV infection induces a long-lasting immune response that protects against homotypic serotypes but not against heterotypic serotypes, which can cause severe disease in secondary infections^3,4^. Anti-DENV antibodies (Abs) produced during primary infections are thought to contribute to secondary heterotypic infection by promoting efficient uptake of Ab–DENV immune complexes by cells bearing the Fc receptor, such as monocytes and macrophages^5^. This process, which is known as Ab-dependent enhancement (ADE), may explain why severe disease is more frequently observed in secondary/subsequent heterotypic DENV infections. The potential to induce ADE also causes problems for the development of antibody therapies and vaccines. For example, approval of the most advanced DENV vaccine that has yet been developed was granted with major restrictions because, compared with placebo, it increased the risk of subsequent occurrence of severe dengue fever in individuals who had not been infected with DENV prior to vaccination, suggesting ADE^6^. Therefore, it is important to evaluate the ability of potentially therapeutic Abs or post-vaccination antisera to protect against or enhance DENV infections. Several mouse models have been developed to understand the pathogenesis of DENV infection and evaluate therapeutics. Mouse-adapted DENV-2 D2S10^7^ and S221 strains^8^, non-adapted DENV-3 C0360/94^9^ and P12/08 strains^10^, and the DENV-4 TVP-376 strain^11^ cause acute lethality within 4–6 days, with human-like symptoms such as thrombocytopenia, increased vascular permeability, and cytokine storms, but without neurologic disease, in interferon (IFN)-α/β–γ receptor (R) double-knockout (dKO) mice ^12^. ADE was accessed in the mouse model infected with D2S10^13^. Thus, there are currently several mouse models available; however, only a limited number of DENV strains actually cause lethal infections with human-like symptoms^7,9,10^. Therefore, it is difficult to test the effect of Abs or antisera against various DENV serotypes, genotypes, and strains. Notably, most of the Abs that are produced in DENV-infected patients target the prM, E, and NS1 proteins^14^, and Abs against E have crucial (but complex and incompletely understood) roles in the control of virus replication via neutralization or ADE^15^. It may be better to have a simpler mouse model in which DENV E and Abs against E can be tested easily. So far, no study has developed alternative mouse models that focus on E and E-specific Abs.

Here, we describe the construction of a recombinant chimeric flavivirus (DV2ChimV), which carried premembrane (prM) and envelope (E) genes of low passage-number DENV-2 R05-624 clinical isolate in a Japanese encephalitis virus (JEV) backbone. Our results demonstrated the potential of DV2ChimV for evaluation of anti-DENV Abs in vitro and in vivo.

## Results

### Replication of DV2ChimV in cultured cells

**Figure 1:**
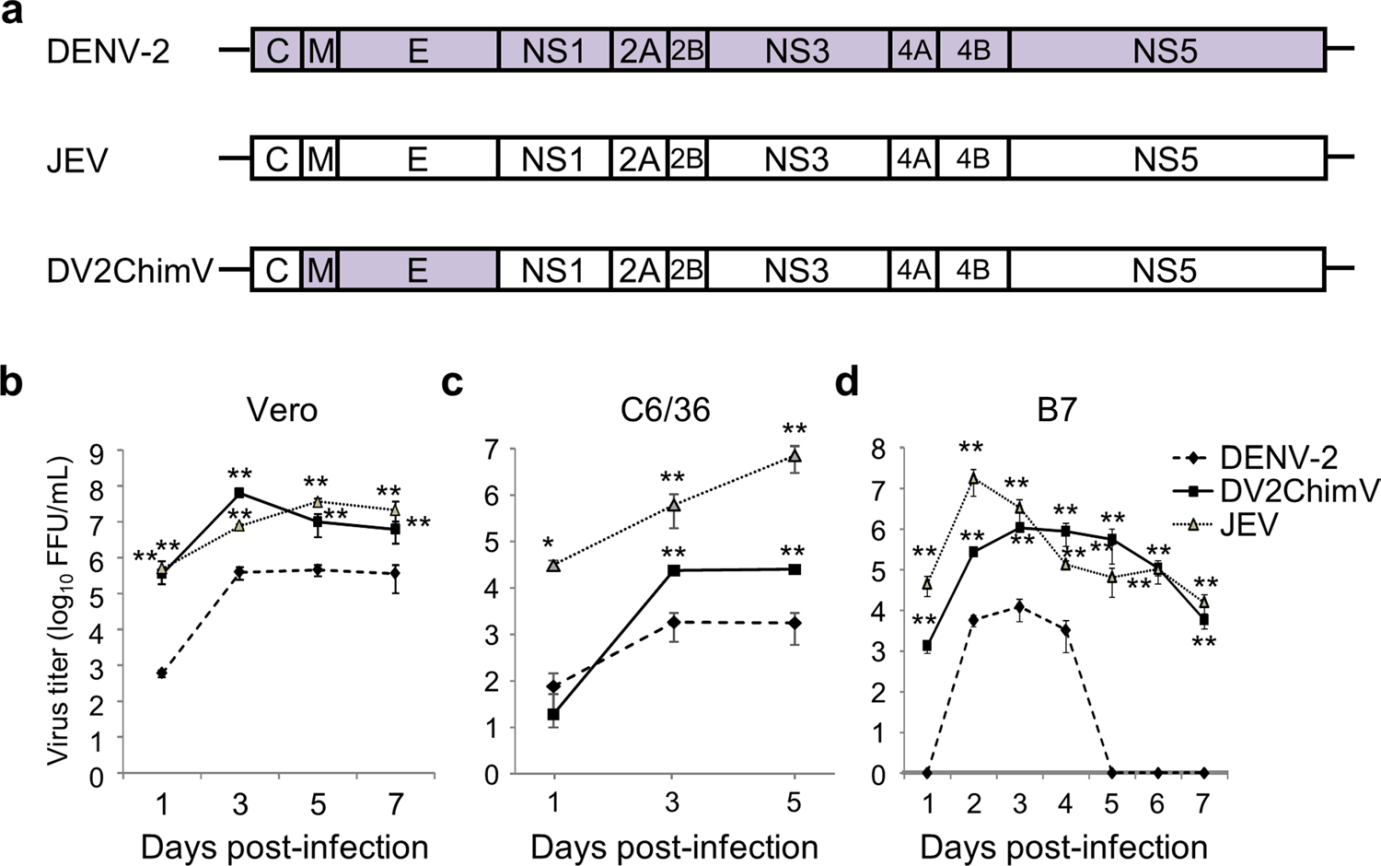
Virus replication in vitro. (**a**) Schematic representation of DENV-2, JEV, and DV2ChimV genomes. (**b**–**d**) Replication of DENV-2, JEV, and DV2ChimV. Mammalian Vero cells, insect C6/36 cells, and mouse B7 cells were infected with DENV-2, JEV, or DV2ChimV. Each cell type was infected at a different multiplicity-of-infection (MOI; 0.01 for Vero and C6/36 cells, 0.2 for B7 cells). Culture media were collected at the indicated time points post-infection. Viral titers were determined by focus-forming assays and expressed as the logarithm of focus-forming units (FFU) per milliliter. Results are expressed as mean + SD of triplicate experiments. Viral titers of each day were analyzed by one-way ANOVA after log transformation. Significance of the levels was assessed by the Dunnett’s Multiple Comparison Test by using viral titer of R05-624 as a control. Statistical differences in viral titers were calculated relative to that of R05-624. *p < 0.01, **p < 0.001.

Replication of DENV-2, JEV, and DV2ChimV was examined in cultured cells (Fig. 1a). Vero (African green monkey kidney) cells infected with DENV-2 R05-624 produced a low level of virus (8.0 × 10^2^ FFU/mL) at Day 1 post-infection (p.i.) and reached a low peak titer (6.3 × 10^5^ FFU/mL) at Day 5 p.i. (Fig. 1b). By contrast, Vero cells infected with DV2ChimV or JEV produced high levels of virus at Day 1 p.i. (4.8 × 10^5^ FFU/mL and 6.8 × 10^5^ FFU/mL, respectively), and reached peak titers of 8.6 × 10^7^ FFU/mL at Day 3 p.i. (DV2ChimV) and 5.0 × 10^7^ FFU/mL at Day 5 p.i. (JEV) (Fig. 1b). In mosquito C6/36 cells, JEV replicated efficiently and reached a peak titer of 5.4 × 10^6^ FFU/mL at Day 5 p.i. (Fig. 1b). Both DV2ChimV and DENV-2 replicated more slowly than JEV, reaching peak titers of 2.0 × 10^4^ FFU/mL at Day 5 p.i. and 1.5 × 10^3^ FFU/mL at Day 3 p.i., respectively (Fig. 1c). Next, we examined the replication of each virus in murine B7 cells derived from wild-type BALB/c mice^16^. In these cells, JEV replicated most efficiently at the early stages, reaching a peak titer of 1.8 × 10^7^ FFU/mL at Day 2 p.i. (Fig. 1d). DV2ChimV also replicated efficiently, reaching a peak titer of 1.1 × 10^6^ FFU/mL. Although production of JEV and DV2ChimV decreased gradually, both maintained production up until at least Day 7 p.i. By contrast, DENV-2 R05-624 started replicating slowly, reaching a peak titer of 1.2 × 10^4^ FFU/mL at Day 3 p.i.; however, virus production completely ceased at Day 5 p.i. (Fig. 1d). DENV-2 production was probably suppressed by the innate immunity in B7 cells because IFN-α and -β in infected B7 cells started to increase on Day 2 p.i and reached a maximum level at Day 5 p.i. (Supplementary Fig. S1). These results demonstrate that DV2ChimV production is similar to that of JEV Nakayama in cultured cells, particularly mouse cells.

### DV2ChimV challenge in mice

**Figure 2:**
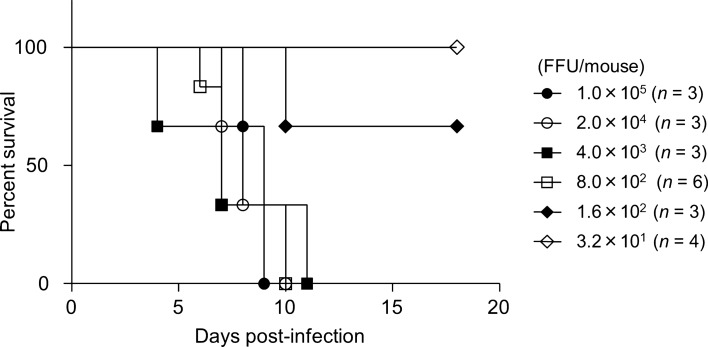
Survival rates of mice infected with chimeric flavivirus. Groups of IFN-α/βR–γR double-knockout mice (8–9 weeks old) were intraperitoneally infected with DV2ChimV at doses ranging from 3.2 × 10^1^ to 1.0 × 10^5^ focus-forming units per mouse (FFU/mouse), and their survival was monitored. Kaplan–Meier survival curves show the percentage of mice surviving at the specified days post-infection. Statistical differences were evaluated by the Log-rank (Mantel–Cox) test. *P* = 0.0077.

DENV-2 R05-624 did not result in lethal infection in any of the mice used in this study (data not shown). DV2ChimV, at a dose of 4.5 × 10^7^ FFU per animal, caused lethal infection in IFN-α/β–γRdKO mice but not in NZBWF1/Slc, C3H/HeSlc, C57BL/6, or IFN-α/βR single-knockout mice (Table 1). When IFN-α/βR–γR dKO mice were intraperitoneally infected with doses of DV2ChimV ranging from 3.2 × 10^1^ FFU to 1.0 × 10^5^ FFU per animal, the lowest dose that resulted in 100% mortality was 8.00 × 10^2^ FFU (LD_50_ = 272 FFU) (Fig. 2).

**Table 1:** Challenge of DV2ChimV in mice.

Mouse	Numbers of mice used for challenge	Percent survival	Time to end point (days)
NZBWF1/Slc	5	100	Asymptomatic survival to 21 days
C3H/HeSlc	5	100	Asymptomatic survival to 21 days
C57BL/6	5	100	Asymptomatic survival to 21 days
IFN-α/βR single KO	3	100	Asymptomatic survival to 21 days
IFN-α/β/γR dKO	5	0	5–7

### Neutralization and ADE assays with DV2ChimV in vitro

**Figure 3:**
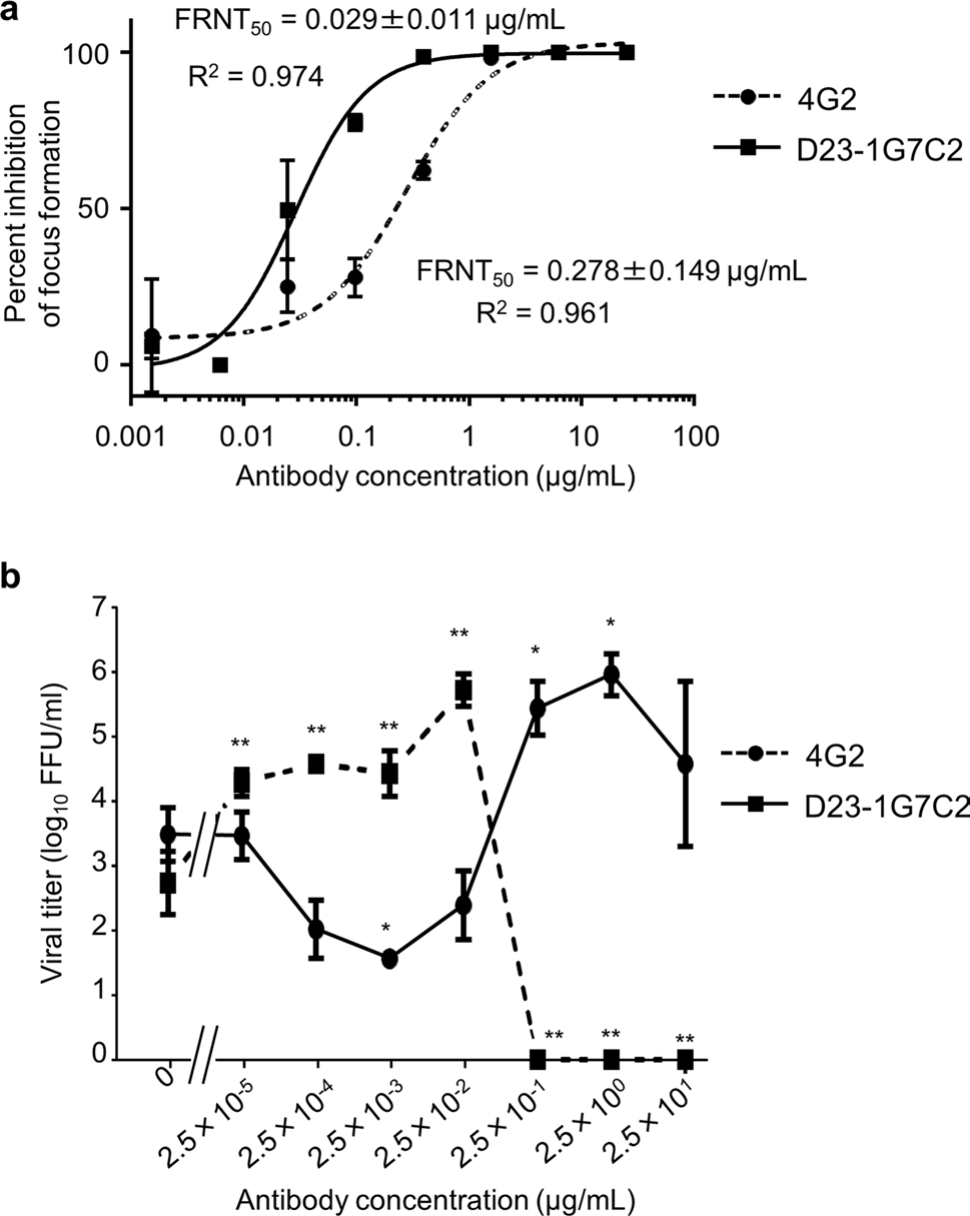
In vitro assessment of antibody-dependent neutralization and enhancement of chimeric flavivirus infection. (**a**) To assess antibody (Ab)-mediated neutralization of virus, mouse monoclonal Ab 4G2 and human monoclonal Ab D23-1G7C2 were each serially diluted to 25, 6.25, 1.56, 0.39, 0.98, 0.024, 0.006, and 0.0015 µg/mL and incubated with 150 focus-forming units (FFU) per well of DV2ChimV. Ab-virus mixtures were then assessed in focus-forming assays to determine the 50% focus reduction neutralization test (FRNT_50_) values of the Abs, which were 0.278 µg/mL for 4G2 and 0.029 µg/mL for D23-1G7C2. FRNT_50_ was calculated from nonlinear log-dose–response curves using GraphPad Prism software. Results are expressed as mean + SD of triplicate experiments. (**b**) To assess Ab-mediated enhancement of infection, 4G2 was serially diluted by tenfold dilution from 2.5 × 10^1^ µg/mL to 2.5 × 10^−5^ µg/mL, and incubated with 4 × 10^4^ FFU of DV2ChimV. Peritoneal-exudate cells were infected with virus–Ab mixtures, at a multiplicity-of-infection of 0.1, and incubated for 3 days. The level of release of viruses into the culture supernatant was determined by titration in Vero cells. Results are expressed as mean + SD of triplicate experiments. Viral titers of each group were analyzed by one-way ANOVA after log transformation. Significance of the levels was calculated relative to Ab untreated by the Dunnett’s Multiple Comparison Test. *p < 0.01, **p < 0.001.

Neutralization assays with cultured cells are commonly used for evaluation of the ability of Abs to protect against pathogens. We examined the neutralizing activity of two Abs to DENV E-protein: human monoclonal Ab (HuMAb) D23-1G7C2, which was previously derived from DENV-infected patients^17^, and the mouse monoclonal Ab 4G2. In the neutralization assay, various concentrations of these Abs were incubated with DV2ChimV, which was then tested in a focus-forming assay in Vero cells. HuMAb D23-1G7C2 demonstrated strong neutralizing activity to DV2ChimV, with a 50% focus reduction neutralization test (FRNT_50_) concentration of 0.029 µg/mL (Fig. 3a). The neutralizing activity of 4G2 was nearly tenfold lower (FRNT_50_ = 0.278 µg/mL).

We next examined whether Abs to DENV E-protein neutralized or enhanced infectivity in mouse macrophages bearing Fc receptors. The 4G2 Ab is known to cause ADE in cultured cells^18^, and D23-1G7C2 has also been shown to have ADE activity in cultured cells^17^. Peritoneal-exudate cells (PECs) were collected from IFN-α/βR–γR dKO mice and infected at a multiplicity-of-infection (MOI) of 0.1 with DV2ChimV, which had been incubated with serially diluted 4G2 or D23-1G7C2. Production of DV2ChimV from infected PECs after 3 days was maximally enhanced (772-fold by D23-1G7C2 and 292-fold by 4G2, compared with viral titers in the absence of Ab treatment) in the presence of 0.025 µg/mL D23-1G7C2 or 2.5 µg/mL 4G2, respectively (Fig. 3b). D23-1G7C20 at > 25 µg/mL reduced viral production, indicating strong neutralization, whereas 4G2 did not neutralize DV2ChimV, even at a concentration of 25 µg/mL, which is consistent with our observation of weaker neutralizing activity than D23-1G7C20 (Fig. 3a). These results demonstrate the potential for the use of DV2ChimV for examination of both neutralizing and enhancing activities of Abs to DENV E-protein.

### Protection and ADE assessment with DV2ChimV in vivo

**Figure 4:**
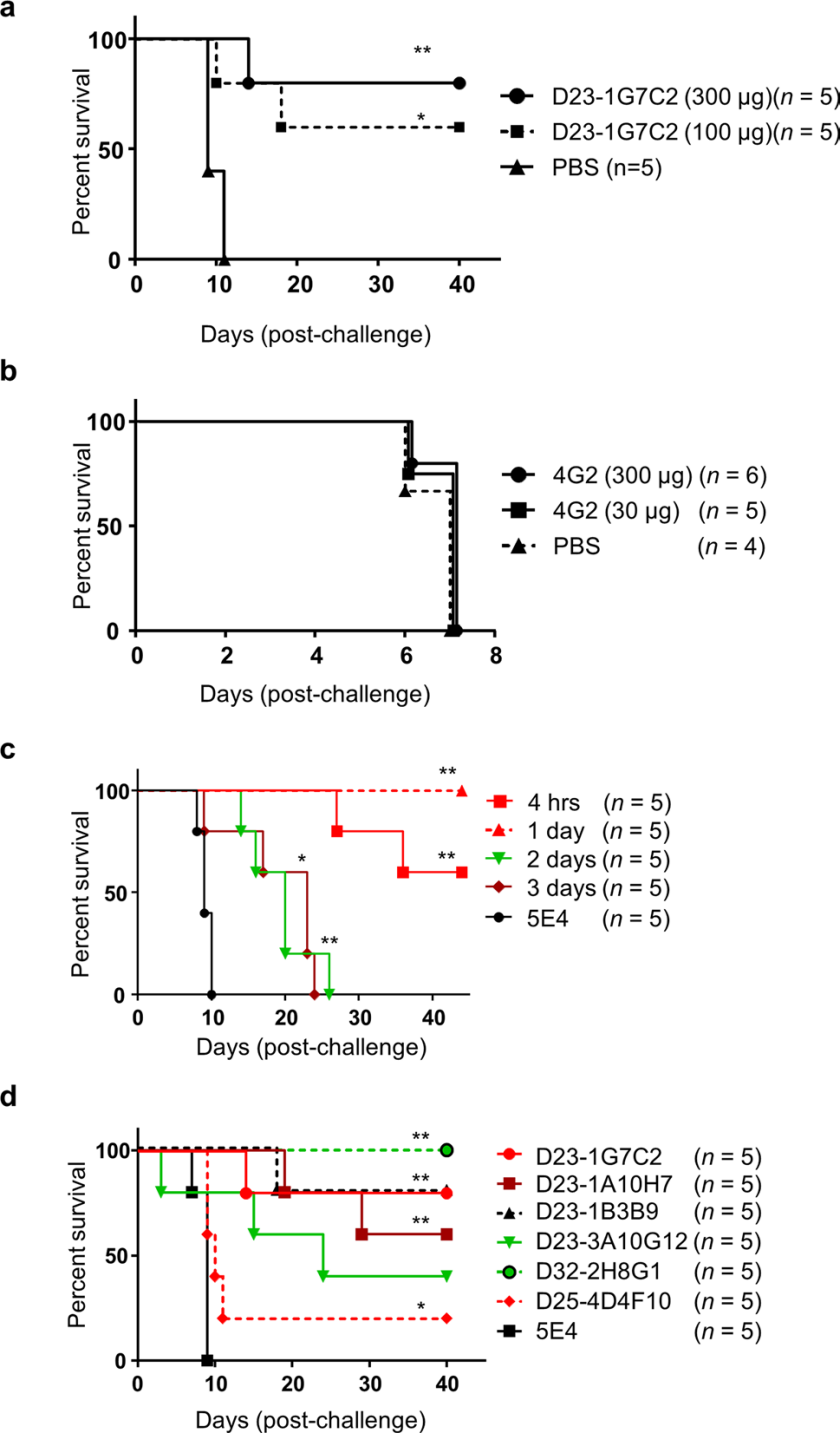
Protection of mice from challenge with chimeric flavivirus by monoclonal antibodies. (**a**) IFN-α/βR–γR double-knockout (dKO) mice (*n* = 5 per group) were intraperitoneally infected with 8.0 × 10^2^ focus-forming units (FFU) per animal of DV2ChimV, followed after 4 h by intraperitoneal injection with 300 µg or 100 µg D23-1G7C2 human monoclonal antibody (HuMAb), or with phosphate-buffered saline (PBS). Statistical differences in survival were observed with D23-1G7C2 relative to PBS. Kaplan–Meier survival curves show the percentage of mice surviving at the specified days post-infection. Statistical differences between individual groups to mock-infected (PBS) control were evaluated by the log rank (Mantel-Cox) test. *P < 0.05, **P < 0.01. (**b**) IFN-α/βR–γR dKO mice (*n* = 4–6 per group) were intraperitoneally infected with 8.0 × 10^2^ FFU per animal of DV2ChimV, followed after 4 h by intraperitoneal injection with 300 µg or 30 µg 4G2 mouse monoclonal antibody, or with PBS. Kaplan–Meier survival curves show the percentage of mice surviving at the specified days post-infection. Statistical differences between individual groups to the mock-infected (PBS) control were evaluated by the log rank (Mantel–Cox) test. No significant difference was found. (**c**) Effect of timing of treatment with HuMAb. IFN-α/βR–γR dKO mice (*n* = 5 per group) were infected with 8.0 × 10^2^ FFU per animal of DV2ChimV and then injected with 300 µg D23-1G7C2 at 4 h, 1 day, 2 days, or 3 days post-infection (p.i.), or with the anti-influenza antibody 5E4 at 4 h p.i. Statistical differences in survival were observed with D23-1G7C2 relative to 5E4. Kaplan–Meier survival curves show the percentage of mice surviving at the specified days post-infection. Statistical differences between individual groups and the isotype IgG (5E4) control were evaluated by the log rank (Mantel–Cox) test. *P < 0.05, **P < 0.01. (**d**) IFN-α/βR–γR dKO mice (*n* = 5 per group) were intraperitoneally infected with 8.0 × 10^2^ FFU per animal of DV2ChimV, followed by intraperitoneal injection with HuMAbs against DENV E-protein (D23-1G7C2, D23-1A10H7, D23-1B3B9, D23-3A10G12, D25-4D4F10, and D32-2H8G1) or against influenza virus (5E4). Statistical differences in survival were observed with anti-E HuMAbs relative to 5E4. Kaplan–Meier survival curves show the percentage of mice surviving at the specified days post-infection. Statistical differences between individual groups to the isotype IgG (5E4) control were evaluated by the log rank (Mantel–Cox) test. *P < 0.05, **P < 0.01.

To determine whether our model system was suitable for evaluation of the ability of Abs to protect against DENV infection, IFN-α/βR–γR dKO mice were infected with DV2ChimV and then injected with Abs to DENV E-protein. For D23-1G7C2, 300 µg administered 4 h p.i. reduced mortality compared with PBS treatment (*P* = 0.0023), with 80% survival at Day 40 p.i. (Fig. 4a). With a 100 µg dose, 60% of the mice were protected at Day 40 p.i. (*P* = 0.0172) By contrast, 4G2 provided no protection (Fig. 4b).

To test its potential for therapeutic application, D23-1G7C2 was introduced at different time points after infection (Fig. 4c). An Ab against influenza virus (5E4) was used as a negative control. Infected IFN-α/βR–γR dKO mice were administered with 300 µg per animal of D23-1G7C2 at 4 h, 1 day, 2 days, and 3 days p.i. (Fig. 4c). D23-1G7C2 provided complete protection (in terms of survival) when it was introduced 1 day p.i., whereas only 60% of mice survived to day 40 p.i. when the Ab was introduced 4 h p.i. Late treatment with Ab 2 days or 3 days p.i. did not prevent mortality, even though it significantly prolonged survival compared with administration of 5E4 at 4 h p.i. (Fig. 4c).

We assessed a number of previously identified HuMAbs to DENV proteins for neutralizing and ADE activities. D23-1A10H7, D23-1B3B9, D23-1G7C2, and D23-3A10G12 are anti-E Abs, and D25-4D4F10 is an anti-prM Ab^19^. D32-2H8G1 has neutralizing activity to DENVs, but its target protein has not been identified^19^. These Abs showed 50% neutralizing activity against DENV-2 (at concentrations ranging from 1.2 to 5.1 μg/mL)^17^. For each Ab, 300 µg was introduced into DV2ChimV-infected IFN-α/βR–γR dKO mice at 4 h post-infection. D32-2H8G1, D23-1B3B9, and D23-1G7C2 provided significant protection, with ≥ 80% survival 40 days p.i., compared with 100% mortality by day 9 p.i. with 5E4 (all *P* < 0.01) (Fig. 4d). Notably, D32-2H8G1 protected 100% of mice, which suggests that it targets DENV prM or E. However, we were unable to identify the target protein of D32-2H8G1 because the Ab did not react with recombinant prME on western blots^17^, suggesting that it might recognize a structurally-specific epitope of the DENV viral particle. The D23-1A10H7 anti-E Ab also provided significant protection, with 60% survival at Day 40 p.i. By contrast, D23-3A10G12 showed only 40% survival at Day 40 p.i. The anti-prM Ab D25-4D4F10 provided only limited protection relative to 5E4 (*P* < 0.05), which is consistent with previous findings^18^. Anti-prM Abs do not usually show strong neutralizing or protective activity. Our observations suggest that the mouse model system is adequate to evaluate the protective abilities of anti-DENV prM and E Abs.

**Figure 5:**
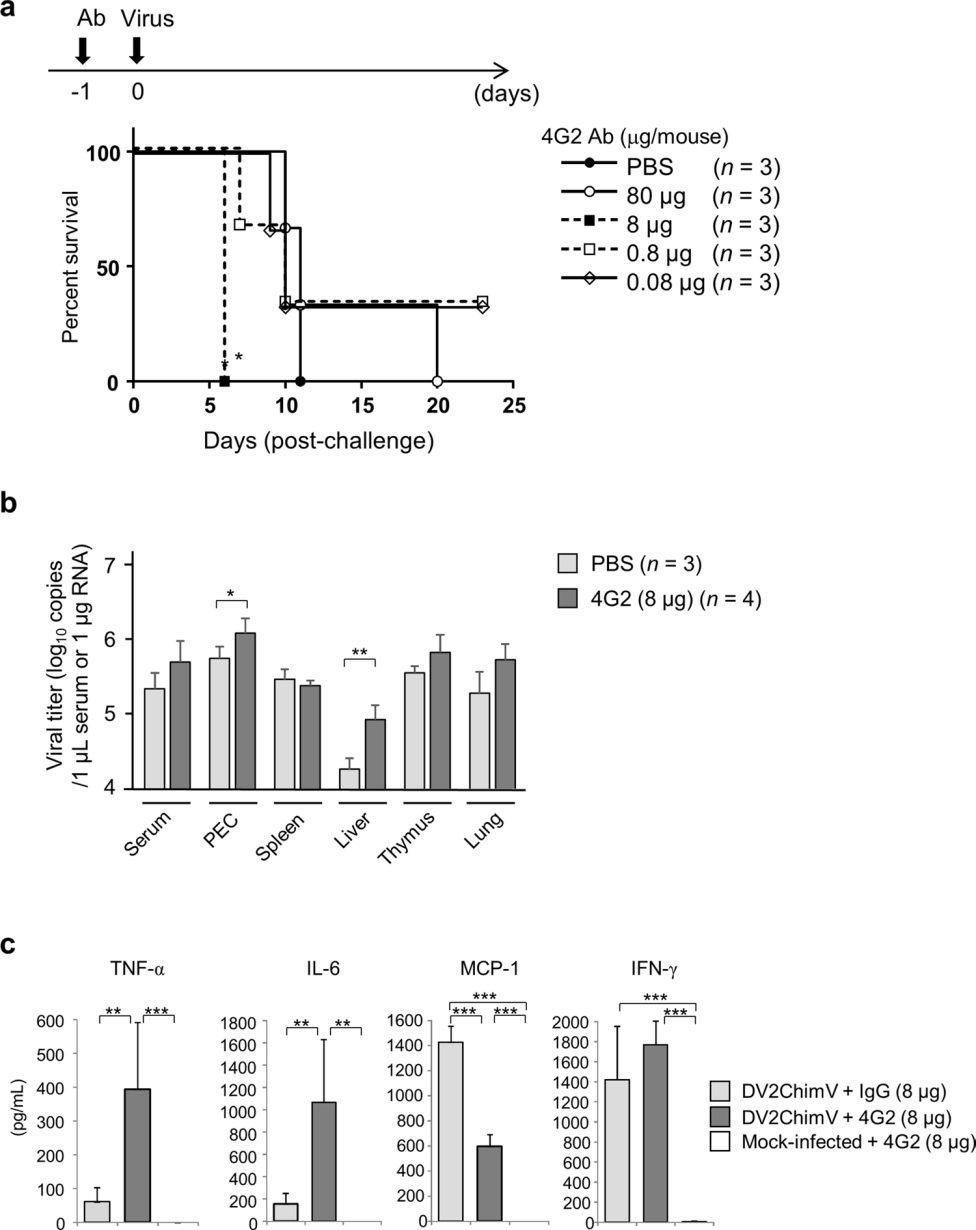
Antibody-dependent enhancement (ADE) in mice. (**a**) IFN-α/βR–γR dKO mice (*n* = 3 per group) were inoculated with 0.08–80 µg per animal of the mouse monoclonal antibody (Ab) to dengue virus E-protein, 4G2, 24 h prior to infection with 8.0 × 10^2^ focus-forming units (FFU) of DV2ChimV chimeric flavivirus. Kaplan–Meier survival curves show the percentage of mice surviving at the specified days post-infection, and were analyzed by the log rank (Mantel–Cox) test. Survival rates were significantly different between mice administered with 8 µg 4G2 and those treated with PBS (*P < 0.05). (**b**) Mice treated with 8 µg 4G2 (*n* = 4) and those treated with PBS (*n* = 3) were euthanized under anesthesia at day 5 p.i., and viral levels in tissues were measured by qRT-PCR. The graph shows mean values and standard deviation. Statistical differences in viral titers in each organ between mice treated with 4G2 and those treated with PBS were calculated using the Unpaired T-test after log transformation. *p < 0.05, *p < 0.01 (**c**) Levels of inflammatory cytokines in the serum of treated mice. IFN-α/βR–γR dKO mice were inoculated with 80 μg 4G2 (*n* = 4) or 80 μg control IgG (*n* = 3) 24 h prior to infection with 8.0 × 10^2^ FFU of DV2ChimV. IFN-α/βR–γR dKO mice were inoculated with 80 μg 4G2 and mock-infected (*n* = 5). At Day 5 p.i., serum samples were collected for analysis by flow cytometry. Levels of TNF-α, IL-6, MCP-1, and IFN-γ in serum samples were determined with the Mouse Inflammation Cytometric Bead Array Kit. Results are expressed as mean + SD of triplicate experiments. Levels of cytokines and chemokines were analyzed by one-way ANOVA. Significance of the levels was assessed by Tukey’s Multiple Comparison Test. **P < 0.01; *** *P* < 0.001.

Next, we used the mouse model to assess ADE. The mouse monoclonal anti-E Ab 4G2 was serially diluted and injected into IFN-α/βR–γR dKO mice 1 day prior to infection with 8.0 × 10^2^ FFU per animal of DV2ChimV. Notably, whereas the mice that received PBS rather than 4G2 all died by day 11 p.i., mice inoculated with 8 µg 4G2 died significantly earlier, by day 6 p.i. (*P* < 0.05), suggesting ADE (Fig. 5a). There were no significant differences in the survival of mice injected with other doses of 4G2 compared with the PBS-treated mice. In addition, IFN-α/βR–γR dKO mice were inoculated with 8 µg 4G2 (8.0 × 10^2^ FFU per animal) at 24 h post-infection with DV2ChimV. These mice died significantly earlier (by Day 7 p.i.) than mice inoculated with control IgG (Supplementary Fig. S2). In similar experiments involving various doses of D23-1G7C2, no induction of ADE was observed (data not shown), presumably because D23-1G7C2 has greater neutralizing activity than 4G2 (Fig. 3a). To further investigate ADE in this system, we assessed viral production in the organs of mice inoculated with 8 µg 4G2 or with PBS, 5 days p.i. Notably, with the exception of PEC and liver, viral production was not significantly higher in the organs of 4G2-treated animals than in those of phosphate-buffered saline (PBS)-treated animals, although there was a trend for increased viral production in serum, PEC, thymus, and lung (Fig. 5b).

Elevated cytokine levels have been observed in DENV-infected patients^20–23^. On Day 5 p.i., TNF-α levels in sera from infected mice treated with 8 μg 4G2 were 6.4-fold higher than those in sera from control infected mice treated with IgG (393.8 pg/mL versus 61.4 pg/mL, respectively), whereas IL-6 levels were 6.9-fold higher (1064.5 pg/mL versus 153.9 pg/mL, respectively) (Fig. 5c). By contrast, the serum level of MCP-1 was 2.4-fold higher in control mice than in mice under ADE conditions (593.4 pg/mL versus 1,425.0 pg/mL, respectively). Induction of IFN-γ was similar in both groups (Fig. 5c). We did not observe detectable levels of IL-12p70 or IL-10 (data not shown). Cytokine levels in mock-infected mice treated with 8 μg 4G2 were measured to establish basal levels. These results suggest that induction of pro-inflammatory cytokines may be important for lethality in this mouse model of ADE.

## Discussion

In this study, we demonstrated the potential of a model system for evaluation of Abs to DENV E-protein. In vivo testing of the efficacy of Abs is carried out in mouse models, but only a limited number of DENV strains cause death in these models at an early time point (< 12 days) with human-like symptoms, such as increased vascular permeability^7,9,10^. Although high doses of some other DENV strains cause lethal infections in IFN-α/βR–γR dKO mice, most of these deaths are caused by the spread of virus into the brain at a late stage after clearance of virus from other organs^10,24^, which does not resemble the pathology of severe dengue fever in humans. Notably, our chimeric virus, DV2ChimV caused death at an early time point, and induced vascular permeability, especially in the liver and intestine at the moribund stage (data not shown).

A benefit of our chimeric virus model is that the E gene in DV2ChimV can be readily replaced with any DENV E sequence of interest, such as that of a currently prevalent strain, to test relevant Abs or antisera. DENVs include several genotypes and strains, which can vary in the sequence of the E gene^25–27^, thereby possibly affecting Ab interactions^25,28^. In addition, there were differences between the characteristics of the highly passaged laboratory DENV strain and those of the low-passaged DENV strain. For example, autologous patient-derived DENVs, but not highly passaged laboratory virus strains, showed a low level of ADE^25,29^. Furthermore, it has been previously reported that different types of cells show different susceptibilities to infection by low-passage DENVs^29^. These observations suggest that there is difference in envelope protein between low-passaged clinical isolates and highly-passaged laboratory strains, although it is still unclear how much this difference between strains or passage number will affect the development of a vaccine and therapeutics, as well as the study of DENV pathogenesis. Another advantage of DV2ChimV is that the JEV-derived backbone, containing C and NS1–5 genes, resulted in a higher level of replication than was seen with DENV-2 in mouse cells, as well as a high level of virulence in mice. Although several mouse models of DENV infection have been reported, high doses of viruses are required^7,9,10^. In the case of human infection, only a small amount of virus is thought to be required for infection^30,31^. In our model, only a few hundred DV2ChimV virions were required for lethal infection (Fig. 2), suggesting that the replication of this virion is faster than that of DENVs in other mouse models. This property implies a requirement of more strict condition for testing effective therapeutics. Neutralizing antibody must efficiently inactivate viral particles, because otherwise the virus can escape to produce large amounts of virus progeny. The efficient replication of DV2ChimV in our model may mirror the high infectivity of DENV in human. Evaluation of Abs in vivo can provide more information than in vitro assessment. For example, five HuMAbs (D23-1G7C2, D23-1A10H7, D23-1B3B9, D23-3A10G12, and D32-2H8G1), which were previously found to have similar neutralizing activity in Vero cells (FRNT_50_ = 1.2 to 5.1 μg/mL to DENV-2)^17^, had considerably different protective effects in our in vivo study (Fig. 4d). In vivo assays could help differentiate between Abs on the basis of characteristics such as stability, effective recycling, and Ab-dependent cell-mediated cytotoxicity. Another potential advantage of our in vivo system was that it enabled observation of the mice over a long period, which made the differences among the Abs more apparent.

By making use of DV2ChimV, we have obtained important information about the target viral protein of D32-2H8G1, which protected 100% of five mice over a 40-day observation period (Fig. 4d). The target protein of D32-2H8G1 had not been previously identified because the Ab did not react with recombinant DENV E-protein^17^, although it did react with cells infected with DENV-2. However, our results suggest that D32-2H8G1 reacts with DENV E because it protected mice from DV2ChimV. Fibriansah et al. identified another potent neutralizing antibody (5J7) that binds across three surface DENV E proteins^32^. D32-2H8G1 may similarly recognize conformationally specific E-protein epitopes located on the surface of the viral particle, which may explain why D32-2H8G1 does not react with recombinant DENV E-protein.

Our results revealed important insights into the role of timing in the effectiveness of Ab treatment. Overall, treatment with D23-1G7C2 was more effective the earlier it was administered post-infection (Fig. 4c). However, there was a notable exception, in that treatment 4 h p.i. resulted in 60% survival, whereas treatment 1 day p.i. resulted in 100% survival. This result suggests that treatment with Ab at a very early time point might attenuate the therapeutic effect. One possible mechanism for attenuation would be the delay or failure of induction of the host immune response. Further study is needed to determine whether immediate treatment after infection results in a delay of the host immune response.

The ADE phenomenon provides a plausible explanation for the occurrence of severe disease in secondary and serial infections^5^. In vitro results demonstrated that appropriate concentrations of anti-E Abs can result in elevation of viral production in cells bearing Fcγ receptors^25,33,34^. In our study, enhancement of viral production by 4G2 ADE was limited in the in vivo mouse model (Fig. 5b) compared with the in vitro murine PEC system (Fig. 3b). Nevertheless, the in vivo effect of ADE on disease outcome was evident in the early deaths of affected mice (Fig. 5a), and in the induction of production of TNF-α and IL-6 (Fig. 5c). It is not yet possible to say whether ADE affects disease outcomes through small changes in viral production that in turn have greater effects on induction of pro-inflammatory cytokines. Other mechanisms might be involved in pathogenesis, such as stimulation of immune responses and pro-inflammatory cytokine production by Abs or Ab-virus complexes. It may be better to consider ADE of infection and ADE of disease separately^35^, although high viral titer is certainly necessary for severe disease^36^.

Recently, Raut et al., reported that DENV virions produced in human are more infectious and mature than those produced in cultured cells^37^. They suggested that there is a structural difference between human plasma and cell-culture derived virions, that is, virions produced in humans contain less undigested prM protein. We measured the number of infectious virions of DV2ChimV in a focus assay and the number of viral genomes by quantitative RT-PCR. DV2ChimV harvested from C6/36 cells contained more infectious viral particles than R05-624, and DV2ChimV harvested from Vero cells contained similar numbers of infectious viral particles to those of DENV-2 R05-624, although JEV contained higher numbers of infectious viral particles (Supplementary Fig. S3). The average number of infectious DV2ChimV virons produced in PEC derived from IFN-α/βR–γR dKO mice was higher than that of DENV-2 virions, although the difference was not significant (Supplementary Fig. S3). Besides, there was no significant difference between the number of infectious DV2ChimV virions and that of JEV virions in sera derived from IFN-α/βR–γR dKO mice. We also examined the levels of E and prM proteins by western-blotting analysis with anti-E (D23-1G7C2) and anti-prM (D25-4D4F10) Abs. DV2ChimV, DENV-2, and JEV were produced from C6/36 cells, Vero cells, and PEC derived from IFN-α/βR-γR dKO mice, and collected. An equivalent number of FFU (5 × 10^5^ ffu) was precipitated by methanol/chloroform, and subjected to western-blotting analysis without reducing agent. An anti-E (D23-1G7C2) does not react to E protein in the presence of reducing agents. In both C6/36 cells and Vero cells, DV2ChimV contained similar levels of uncleaved prM protein to those of original DENV-2 R05-624 (Supplementary Fig. S4). Two bands of DV2ChimV and DENV-2 E proteins may be due to the result of different levels of glycosylation or partial cleavage. Besides, dimerized E proteins were found because E protein forms dimer through disulfide bonds. Interestingly, DV2ChimV produced in PEC derived from IFN-α/βR–γR dKO mice contained lower levels of uncleaved prM (Supplementary Fig. S4), although we failed to detect E or prM in the serum of this model mouse. PEC is a major target cell in this model (data not shown). On the other hand, JEV viral particles appeared to contain less uncleaved prM; however, this may be due to the low reactivity of anti-prM Ab. In terms of maturation, DV2ChimV may produce mature virions at a higher rate than DENV-2 in IFN-α/βR–γR dKO mice. However, it is still unclear whether virion particle heterogeneity influences DENV serotypes, genotypes, or strains^38^. So far, there has been no report about the degree of maturity of DENV in mice. Further study is needed to understand the effect of viral maturation.

There is a question about whether this model using a chimeric dengue virus is applicable to the study of the pathogenesis of severe dengue. DENV NS1 protein is thought to play an important role^39–41^; however, the detailed mechanism is not fully understood. The NS1 gene of DV2ChimV is derived from the JEV genome. However, DV2ChimV infection caused vascular leakage and thrombocytopenia in IFN-α/βR–γR dKO mice (data not shown). Further comparisons of DV2ChimV and DENV would provide more information about pathogenesis.

Our results demonstrated the potential of a chimeric flavivirus for the evaluation of therapeutic Abs to DENV. Although humans and mice differ physiologically, we believe that this system will be useful for early evaluation of potentially therapeutic Abs. It should also be amenable to the assessment of antisera resulting from the use of vaccine candidates. Although we believe that the chimeric flavivirus and other recombinant viruses will be useful for the development of therapeutics, we recommend that experiments using chimeric viruses be conducted under the proper degree of biological containment to avoid accidental release.

## Methods

### Construction of the chimeric virus DV2ChimV

**Figure 6:**
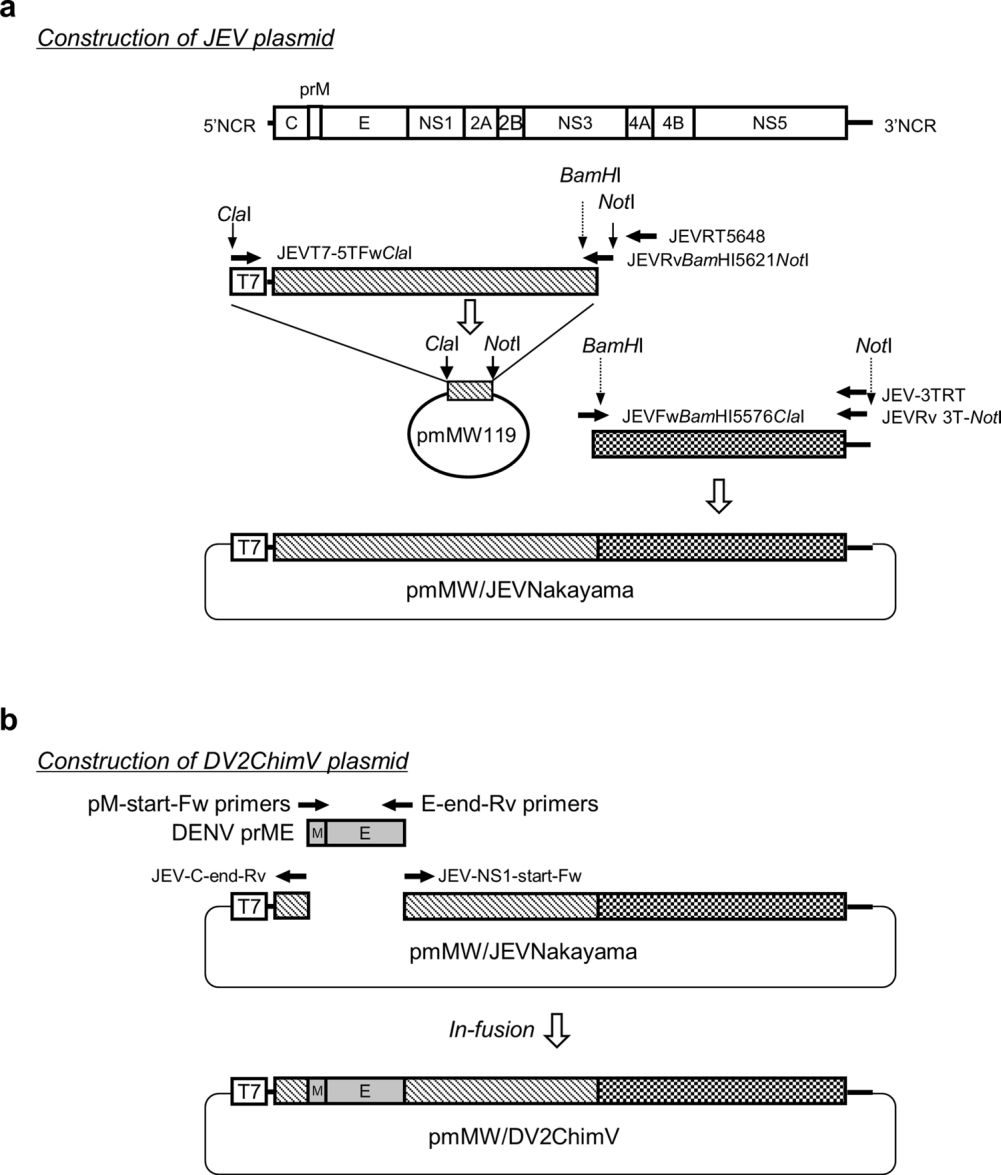
Schematic representation of the strategy used to construct the recombinant plasmids pmMW/JEVNakayama and pmMW/DV2ChimV. (**a**) pmMW/JEVNakayama was constructed by insertion of the Japanese encephalitis virus (JEV) genome and the T7 promoter sequence into pmMW119 between the ClaI and NotI sites. (**b**) The premembrane (prM) and envelope (E) gene sequences of pmMW/JEVNakayama were replaced with the corresponding DENV-2 R05-624 prME gene sequences.

To construct DV2ChimV, an infectious cDNA clone of the JEV strain Nakayama, plasmid pmMW/JEVNakayama was first constructed (Fig. 6a). JEV Nakayama RNA was extracted from culture fluid with a QIAamp Viral RNA Mini Kit (Qiagen, Hilden, Germany), in accordance with the manufacturer’s instructions. To synthesize viral cDNA, reverse transcription was performed with SuperScript III Reverse Transcriptase (Invitrogen, Carlsbad, CA, USA) and the following NS3-specific primers: JEVRT5648 (5′-CCACTGCTCCATGCCCTGTCTGG-3′) for the 5′-terminal region and JEV-3TRT (5′-AGATCCTGTGTTCTTCCTCACCAC-3′) for the 3′-terminal region. The NS3–5′-terminal region of the JEV Nakayama genome was amplified with primers JEVT7-5TFw*Cla*I (5′-GGCATCGATTAATACGACTCACTATAGAGAAGTTTATCTGCGTGAACTTCTTGGC-3′), which contained the T7 polymerase promoter sequence, and JEVRv*Bam*HI5621*Not*I (5′-GGCGCGGCCGCCATCTTGCAAATCATGGATTGGGG-3′) by PCR with high-fidelity PrimeSTAR GXL DNA Polymerase (Takara, Otsu, Japan). The NS3–3′-terminal region of the JEV Nakayama genome was amplified with primers JEVFw*Bam*HI5576*Cla*I (5′-GGCATCGATGACAGCGACCCCGCCTGGAACCAC-3′) and JEVRv 3 T-*Not*I (5′-GCCGCGGCCGCAGATCCTGTGTTCTTCCTCACCAC-3′). The PCR product of the 5′ region was cloned into pmMW119^42^ between the *Cla*I and *Not*I sites, and the resultant plasmid was named pmMW119JEV/Nakayama5′-NS3. A complete recombinant JEV Nakayama clone was constructed by insertion of the PCR fragment of the 3′ region into pmMW119JEV/Nakayama5′-NS3.

The infectious clones of pmMW/JEVNakayama and the previously constructed DENV-2 pmMW/R05-624^42^ were used as DNA sources for the production of DV2ChimV. The prME sequence of pmMW/JEVNakayama was replaced with the corresponding DENV-2 R05-624 prME gene sequences, resulting in plasmid pmMW/DV2ChimV (Fig. 6b). The prME region of R05-624 was amplified with DV2-pM-start-Fw (5′-GCCTGCGCAGGAGCCTTCCATCTAACCACACGCAACGG-3′) and DV2-E-end-Rv (5′-GGCACATCCAGTGTCGGCCTGCACCATGACTCCCAA-3′), with pmMW/R05-624 as the template. The vector backbone sequence was amplified with JEV-C-end-Rv (5′-GGCTCCTGCGCAGGCTATGATAAC-3′) and JEV-NS1-start-Fw (5′-GACACTGGATGTGCCATTGACGTC-3′), with pmMW/JEVNakayama as the template. PCR fragments were ligated using the In-fusion HD Cloning Kit (Takara). All cloning steps were performed using *Escherichia coli* HB101 competent cells (Takara). All plasmids generated were verified by sequencing using gene-specific primers (Supplementary Table).

### Virus production

To generate chimeric virus, plasmid containing full-length DVChimV sequence was linearized with *Not*I, and purified by phenol–chloroform extraction and ethanol precipitation. Linearized DNA (1 µg) was transcribed with a mMESSAGE mMACHINE Kit (Ambion, Foster City, CA, USA) containing m^7^G(5′)ppp(5′)G cap analogue, in accordance with the manufacturer’s instructions. Co-cultured Vero/C6/36 cells were transfected with 2 µg transcribed RNA and incubated at 37 °C in an atmosphere containing 5% CO_2_ for 3 days^42^.

### Cells

Vero cells (African green monkey kidney) were cultured in Eagle’s minimum essential medium (MEM) (Sigma-Aldrich, St. Louis, MO, USA), and murine B7 cells (derived from wild-type BALB/c mice)^16^ and PECs were cultured in Dulbecco’s modified Eagle medium (DMEM) (Nacalai Tesque, Kyoto, Japan) supplemented with 10% fetal calf serum (FCS) at 37 °C in a humidified atmosphere containing 5% CO_2_.

### Focus-forming assay

Viral titers were determined by measuring focus-forming units (FFU). Briefly, culture media underwent serial tenfold dilution with MEM containing 2% FCS. Vero cells in 96-well microplates were inoculated with diluted media and incubated for 2 h at 37 °C. The cells were then cultured for 72 h with MEM containing 2% FBS and 2% carboxymethylcellulose (overlay medium). Staining of infected cells was performed as previously reported^10^.

### Preparation of PECs

Mice were intraperitoneally injected with 2.5 mL per animal of 4% thioglycolate broth (Becton Dickinson, Franklin Lakes, NJ, USA). After 5 days, PECs were harvested by one lavage of the peritoneal cavity with 5 mL PBS containing 3 mM EDTA. PECs were collected by centrifugation at 800×*g* for 10 min at room temperature and stored at − 80 °C.

### Purification of HuMAbs and 4G2 by protein G affinity column

MAbs were purified from serum-free hybridoma medium (Hybridoma SFM, Life Technologies, Carlsbad, CA, USA) by protein G column chromatography (HiTrap Protein G HP columns, GE Healthcare, Little Chalfont, UK). IgG concentrations were measured with the Pierce BCA Protein Assay Kit (Thermo Fisher Scientific, Waltham, MA, USA).

### Neutralization assay

Four-fold serial dilutions (100, 25, 6.25, 1.56, 0.39, 0.98, 0.024, and 0.006 μg/mL) of 4G2 and D23-1G7C2 were prepared and reacted with DV2ChimV (150 FFU per well) (50 μL Ab + 50 μL virus) for 30 min at 37 °C. The Ab-virus complex was then added to Vero cells, followed by 100 μL overlay medium, and cultured for 3 days. FRNT_50_ values were calculated as described previously^17^.

### In vitro ADE assay

PECs were obtained from thioglycolate-treated IFN-α/βR–γR dKO mice and cultured in DMEM supplemented with 10% FBS. Purified 4G2 and D23-1G7C2 were serially diluted (tenfold from 50 μg/mL to 50 pg/mL), and 100 μL Ab at each concentration was incubated with 100 μL DV2ChimV at 37 °C for 30 min and then used to inoculate PECs, which were then incubated for 3 days at 37 °C. Culture supernatants were then collected for viral titration. Assays were performed in triplicate.

### Ethics statement

All animal experiments were carried out under the applicable laws and guidelines for the care and use of laboratory animals at the Research Institute for Microbial Diseases, Osaka University. The study was approved by the Animal Experiment Committee of the Research Institute for Microbial Diseases, Osaka University (#H25-09-1), as specified in the Fundamental Guidelines for the Proper Conduct of Animal Experiment and Related Activities in Academic Research Institutions under the jurisdiction of the Ministry of Education, Culture, Sports, Science and Technology, Japan, 2006 (http://www.scj.go.jp/ja/info/kohyo/pdf/kohyo-20-k16-2.pdf). Trained laboratory personnel performed anesthesia of mice via intraperitoneal injection of a mixture of medetomidine, midazolam, and butorphanol during viral injection, and euthanasia by cervical dislocation.

### Experiments involving mice

NZBWF1/Slc, C3H/HeSlc, and C57BL/6 mice (4 weeks old) were purchased from SLC (Hamamatsu, Japan), inoculated intraperitoneally with DV2ChimV, and observed until 21 days p.i. Trained laboratory personnel anesthetized the mice by intraperitoneal injection of a mixture of medetomidine, midazolam, and butorphanol prior to viral injection. IFN-α/βR single-knockout and IFN-α/βR–γR dKO (IFN-α/β/γR dKO) mice^10^ were bred and maintained under specific-pathogen-free conditions at the animal facility of RIMD Osaka University. For the experiment to observe protective effects and ADE, 8–10-week-old IFN-α/βR–γR dKO mice were administered a single dose of MAb via the intraperitoneal route, before or after infection. IFN-α/βR–γR dKO mice were challenged with DV2ChimV intraperitoneally. Following infection, mice were observed daily for any conspicuous clinical manifestations. Mice were humanely euthanized, to avoid unnecessary suffering, if they exhibited weight loss > 20% of initial body weight in the 3 day period after infection, or weight loss of 25% in the 7 day period after infection. Mice were euthanized by cervical dislocation under anesthesia.

### Cytometric bead assay

Levels of inflammatory cytokines were determined with the Mouse Inflammation Cytometric Bead Array Kit (Becton Dickinson). Sera were tenfold diluted with Assay Diluent. Diluted samples (50 µL) were incubated with 50 µL mixed Mouse Inflammation Capture Beads and 50 µL Mouse Inflammation PE Detection Reagent for 2 h. After incubation and a single wash with Wash Buffer, beads were detected by flow cytometry. All steps were performed at room temperature. Final concentrations were calculated in pg/mL.

### Quantitative RT-PCR analysis of DV2ChimV RNA

To measure viremia levels, viral RNA (vRNA) was extracted from 70 µL mouse serum with the QIAamp Viral RNA Mini Kit (Qiagen) into 60 µL elution buffer. To quantify vRNA in the spleen, liver, kidney, thymus, lung, brain, PECs, and bone marrow, total RNA was extracted from these organs with TRIzol RNA Isolation Reagents (Life Technologies), with final resuspension into 30 µL RNase-free water. The concentration of each extracted RNA was adjusted to 50 µg/mL. The following primers, modified from a previous report^43^, were used for PCR: JEF (5′-AGAGCGGGGAAAAAGGTCAT-3′) and JER#110 (5′-CTTCACGCTCTTCCTACAGT-3′). One-step, real-time quantitative RT-PCR amplification with SYBR Green I was performed with the CFX Connect Real-Time System (Bio-Rad, Hercules, CA, USA) and the One-Step SYBR PrimeScript RT-PCR Kit II (Takara). The final concentration of each PCR primer was 0.08 µM, and the concentration of total RNA was 8 µg/mL, with 12.5 µL reaction volumes. The conditions for reverse transcription were 42 °C for 5 min and 95 °C for 10 min. PCR amplification used 45 cycles of 95 °C for 5 s, 55 °C for 30 s, and 72 °C for 30 s. The quantity of vRNA in the initial total RNA was determined by interpolation analysis from a standard curve generated from tenfold serial dilutions of in vitro-transcribed DV2ChimV RNA made with the MEGAscript Kit (Ambion). The limit of detection was ≥ 10 copies. Data were analyzed with CFX Manager ver. 1.6 (Bio-Rad). To quantify vRNA derived from organs, the amounts were normalized to the total RNA from corresponding organs of mock-infected mice.

### Data analysis

All data were analyzed with Graphpad Prism software (Graphpad, San Diego, CA, USA).

## Supplementary Information

Supplementary Information.
