# Supplementary material for: Chimeric flavivirus enables evaluation of antibodies against dengue virus envelope protein in vitro and in vivo
Source: Sci Rep. 2020 Dec 9;10:21561. doi: 10.1038/s41598-020-78639-x (PMC7725774; doi:10.1038/s41598-020-78639-x)
Supplement: Supplementary file 1 — Supplementary Information. [file 41598_2020_78639_MOESM1_ESM.pdf]

Chimeric flavivirus enables evaluation of antibodies against dengue virus envelope protein *in vitro* and *in vivo*

Takeshi Kurosu[a,c,*], Keiko Hanabara[a], Azusa Asai[a], Sabar Pambudi[a], Supranee Phanthanawiboon[a], Magot Diata Omokoko[a], Ken-ichiro Ono[b], Masayuki Saijo[c,] , Pongrama Ramasoota[d], and Kazuyoshi Ikuta[a]
[a]Research Institute for Microbial Diseases (RIMD), Osaka University, Suita, Osaka, 565-0871, Japan
[b]Medical and Biological Laboratories CO., LTD., Ina, Nagano, 396-0002, Japan
[c]Department of Virology I, National Institute of Infectious Diseases, 4-7-1 Gakuen, Musashimurayama-shi, Tokyo, 208-0011, Japan
[d]Center of Excellence of Antibody Research, Department of Social and Environmental Medicine, Faculty of Tropical Medicine, Mahidol University, Bangkok 10400, Thailand

Name of primers	Sequence
JEVseq1(301-321)	GGAAAGCAGTGGAAAAGAGTG
JEVseq2(812-832)	GGATTCAACGAAAGCCACACG
JEVseq3(1320-1340)	ACATGTGCAAAATTCTCCTGC
JEVseq4(1851-1871)	GACAAACTGGCTCTGAAAGGC
JEVseq5(2381-2400)	ACTCTGGATGGGCGTCAACG
JEVseq6(2901-2920)	GAATGCCCTGATGAGCACAG
JEVseq7(3432-3451)	CCGCCCCTACGATTCCGGAC
JEVseq8(3970-3989)	CAACCTCCTCCGTCACCATG
JEVseq9(4490-4509)	TGTTCCATGGAAGGTCTGGG
JEVseq10(5062-5081)	CAATGGAGTTGAGCTTGGCG
JEVseq11(5588-5607)	TGACTCAAATGCCCCAATCC
JEVseq12(6112-6131)	CCAATGGACTGGTGGCCCAG
JEVseq13(6650-6672)	GATGACAGGAGGATTCTTTCTAC
JEVseq14(7203-7222)	GTCTTCCTTGGCTGTTGGGG
JEVseq15(7760-7779)	GAGAGAGGCCATAATCGAGG
JEVseq16(8320-8339)	CCAATCACGAGATGTATTGG
JEVseq17(8866-8885)	CCAAGGAAGAATTCATAAAG
JEVseq18(9401-9420)	CAGGCACAAAGTGGTCAAGG
JEVseq19(9928-9946)	GGAATGTGAAGGACACAGC
JEVseq20(10473-10492)	AGCTGCCACCGGATACTGGG
SeqMWFw2204	TACGCCAGCTGGCGAAAGG
SeqMWRv2398	GCTTCCGGCTCGTATGTTG
DV2Seq(1044-1063)	AAAACAAACCAACATTGGATT
DV2Seq(1620-1640)	RTCAAATTGGATACAGAAAGA

Supplementary Table. Primers used for full-length pmMW/JEVNakayama and pmMW/DV2ChimV plasmids.

Chimeric flavivirus enables evaluation of antibodies against dengue virus envelope protein *in vitro* and *in vivo*

Takeshi Kurosu[a,c,*], Keiko Hanabara[a], Azusa Asai[a], Sabar Pambudi[a], Supranee Phanthanawiboon[a], Magot Diata Omokoko[a], Ken-ichiro Ono[b], Masayuki Saijo[c,] , Pongrama Ramasoota[d], and Kazuyoshi Ikuta[a]
[a]Research Institute for Microbial Diseases (RIMD), Osaka University, Suita, Osaka, 565-0871, Japan
[b]Medical and Biological Laboratories CO., LTD., Ina, Nagano, 396-0002, Japan
[c]Department of Virology I, National Institute of Infectious Diseases, 4-7-1 Gakuen, Musashimurayama-shi, Tokyo, 208-0011, Japan
[d]Center of Excellence of Antibody Research, Department of Social and Environmental Medicine, Faculty of Tropical Medicine, Mahidol University, Bangkok 10400, Thailand

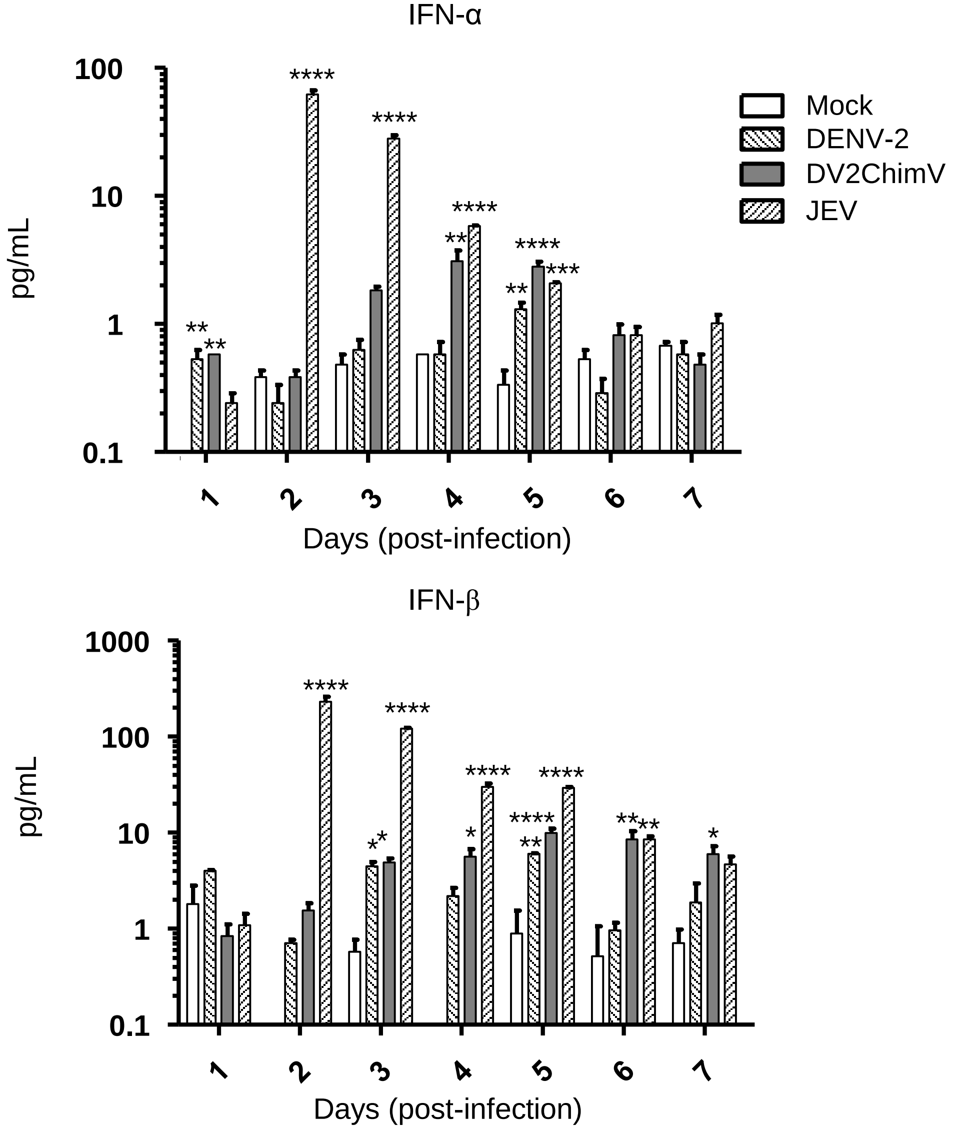

Supplementary Figure S1.

Productions of IFN-α and IFN-β in B7 cell culture medium. B7 cells were infected with mock, DENV-2, DV2ChimV, and JEV. Culture supernatants were collected every day until Day 7 p.i., and the levels of IFN-α or IFN-β were measured using Verikine IFN-α or IFN-β ELISA kits. Results are expressed as mean +SD of triplicate experiments. Productions of IFN-α or IFN-β were analyzed by one-way ANOVA. Significance of the levels relative to that of mock was assessed by the Dunnett's Multiple Comparison Test. *$p < 0.05$, ** $p < 0.01$, *** $p < 0.001$, **** $p < 0.0001$,

Chimeric flavivirus enables evaluation of antibodies against dengue virus envelope protein *in vitro* and *in vivo*

Takeshi Kurosu[a,c,*], Keiko Hanabara[a], Azusa Asai[a], Sabar Pambudi[a], Supranee Phanthanawiboon[a], Magot Diata Omokoko[a], Ken-ichiro Ono[b], Masayuki Saijo[c,] , Pongrama Ramasoota[d], and Kazuyoshi Ikuta[a]
[a]Research Institute for Microbial Diseases (RIMD), Osaka University, Suita, Osaka, 565-0871, Japan
[b]Medical and Biological Laboratories CO., LTD., Ina, Nagano, 396-0002, Japan
[c]Department of Virology I, National Institute of Infectious Diseases, 4-7-1 Gakuen, Musashimurayama-shi, Tokyo, 208-0011, Japan
[d]Center of Excellence of Antibody Research, Department of Social and Environmental Medicine, Faculty of Tropical Medicine, Mahidol University, Bangkok 10400, Thailand

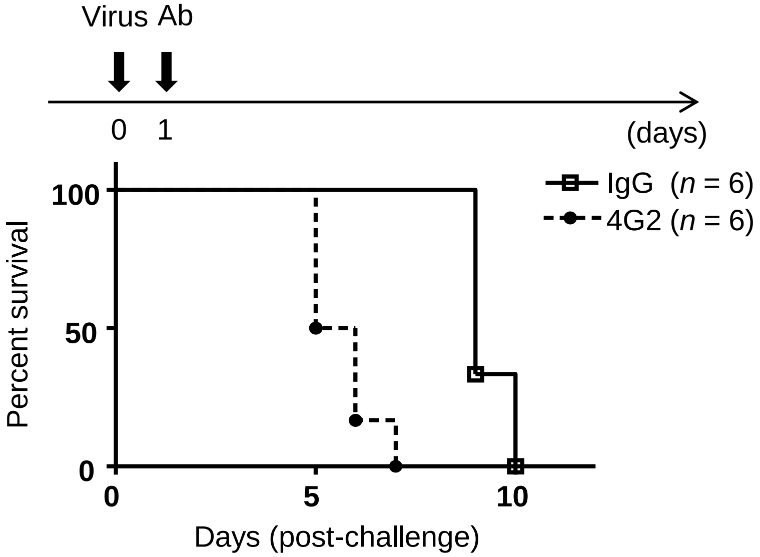

Supplementary Figure S2.
Survival of IFN-α/βR-γR double knockout (KO) mice intraperitoneally infected with 800 focus-forming units (FFU) of DV2ChimV, followed by the intraperitoneal injection with an anti-E Ab (n = 6) or an isotype control Ab (n = 6) on Days 1 p.i. Kaplan-Meier survival curves show the percentage of mice surviving at the specified days post-infection. Statistical differencesbetween anti-E and isotype control treated mice was evaluated by the log rank (Mantel-Cox) test. p = 0.0007

Chimeric flavivirus enables evaluation of antibodies against dengue virus envelope protein *in vitro* and *in vivo*

Takeshi Kurosu[a,c,*], Keiko Hanabara[a], Azusa Asai[a], Sabar Pambudi[a], Supranee Phanthanawiboon[a], Magot Diata Omokoko[a], Ken-ichiro Ono[b], Masayuki Saijo[c,], Pongrama Ramasoota[d], and Kazuyoshi Ikuta[a]
[a]Research Institute for Microbial Diseases (RIMD), Osaka University, Suita, Osaka, 565-0871, Japan
[b]Medical and Biological Laboratories CO., LTD., Ina, Nagano, 396-0002, Japan
[c]Department of Virology I, National Institute of Infectious Diseases, 4-7-1 Gakuen, Musashimurayama-shi, Tokyo, 208-0011, Japan
[d]Center of Excellence of Antibody Research, Department of Social and Environmental Medicine, Faculty of Tropical Medicine, Mahidol University, Bangkok 10400, Thailand

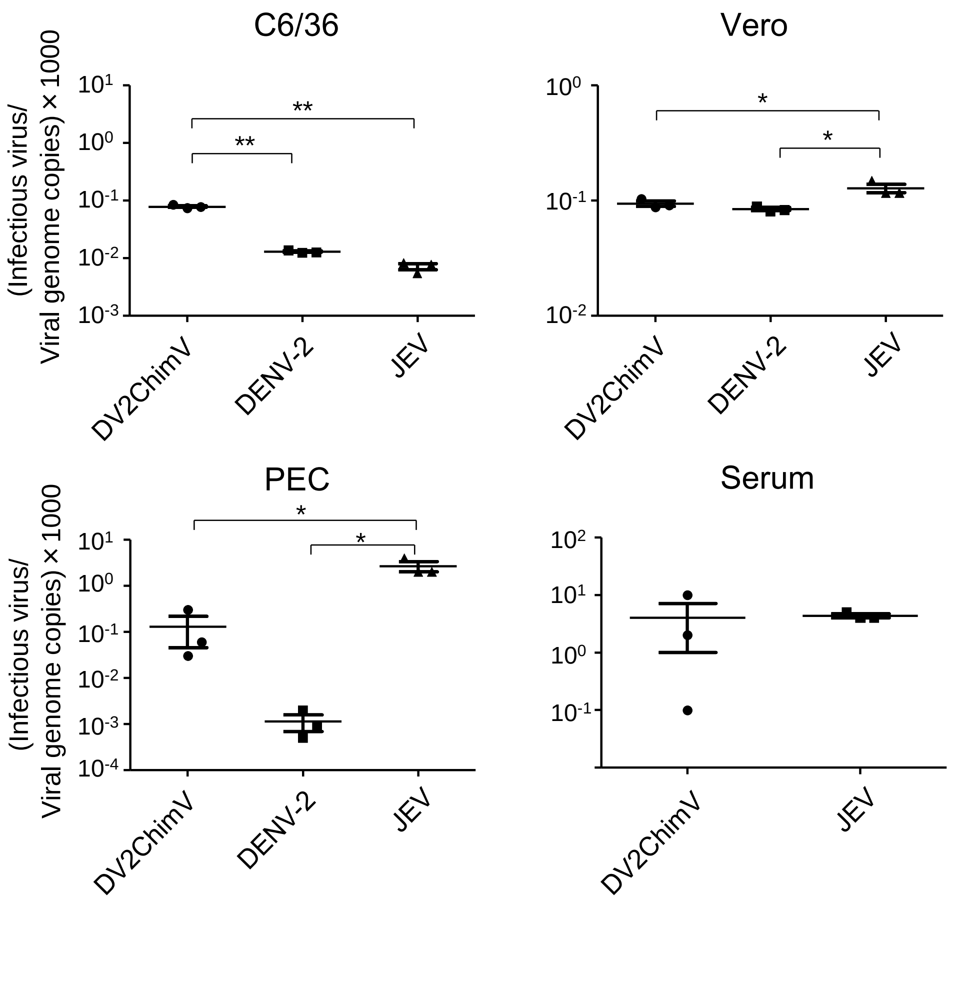

Supplementary Figure S3.
Infectivity of DV2ChimV, DENV-2, and JEV virions produced in C6/36, Vero cells and PEC derived from IFN-α/βR–γR dKO mice. Culture supernatants were collected from each cell, and viral RNA (vRNA) was extracted from 200 μl culture supernatants with High Pure Viral RNA kit (Roche, Indianapolis, IN, USA). Sera were collected from FN-α/βR–γR dKO mice infected with DV2ChimV and JEV, and vRNA was extracted with High Pure Viral RNA kit. The following primers were used for PCR,

primer set 1: DV2E_Fw2 (5'-ACAGGCCTCGACTTCAATGA-3') and DV2E_Rv2 (5'-TGGATTTCTGTGGCTCCTGT-3') for DV2ChimV and DENV-2 or primer set 2: JEF (5′-AGAGCGGGGAAAAAGGTCAT-3′) and JER#110 (5′-CTTCACGCTCTTCCTACAGT-3′) for JEV. One-step, real-time quantitative RT-PCR amplification with SYBR Green I was performed with the LightCycler 96 (Roche). The quantity of vRNA in the initial total RNA was determined by interpolation analysis from a standard curve generated from 10-fold serial dilutions of *in vitro*-transcribed DV2ChimV RNA made with the MEGAscript Kit (Ambion). Viral titer was quantified by a focus-forming assay in Vero cells as described in methods. Viral infectivity in cell culture supernatants (C6/36 and Vero cells) was estimated by the number of infectious virions by the number of genomes in each specimen. Results are expressed as mean +SD of triplicate experiments. Viral titers were analyzed by one-way ANOVA. Significance of the levels was assessed by the Turkey Test. *$p < 0.01$, **$p < 0.001$.

Chimeric flavivirus enables evaluation of antibodies against dengue virus envelope protein *in vitro* and *in vivo*
Takeshi Kurosu[a,c,*], Keiko Hanabara[a], Azusa Asai[a], Sabar Pambudi[a], Supranee Phanthanawiboon[a], Magot Diata Omokoko[a], Ken-ichiro Ono[b], Masayuki Saijo[c,] , Pongrama Ramasoota[d], and Kazuyoshi Ikuta[a]
[a]Research Institute for Microbial Diseases (RIMD), Osaka University, Suita, Osaka, 565-0871, Japan
[b]Medical and Biological Laboratories CO., LTD., Ina, Nagano, 396-0002, Japan
[c]Department of Virology I, National Institute of Infectious Diseases, 4-7-1 Gakuen, Musashimurayama-shi, Tokyo, 208-0011, Japan
[d]Center of Excellence of Antibody Research, Department of Social and Environmental Medicine, Faculty of Tropical Medicine, Mahidol University, Bangkok 10400, Thailand

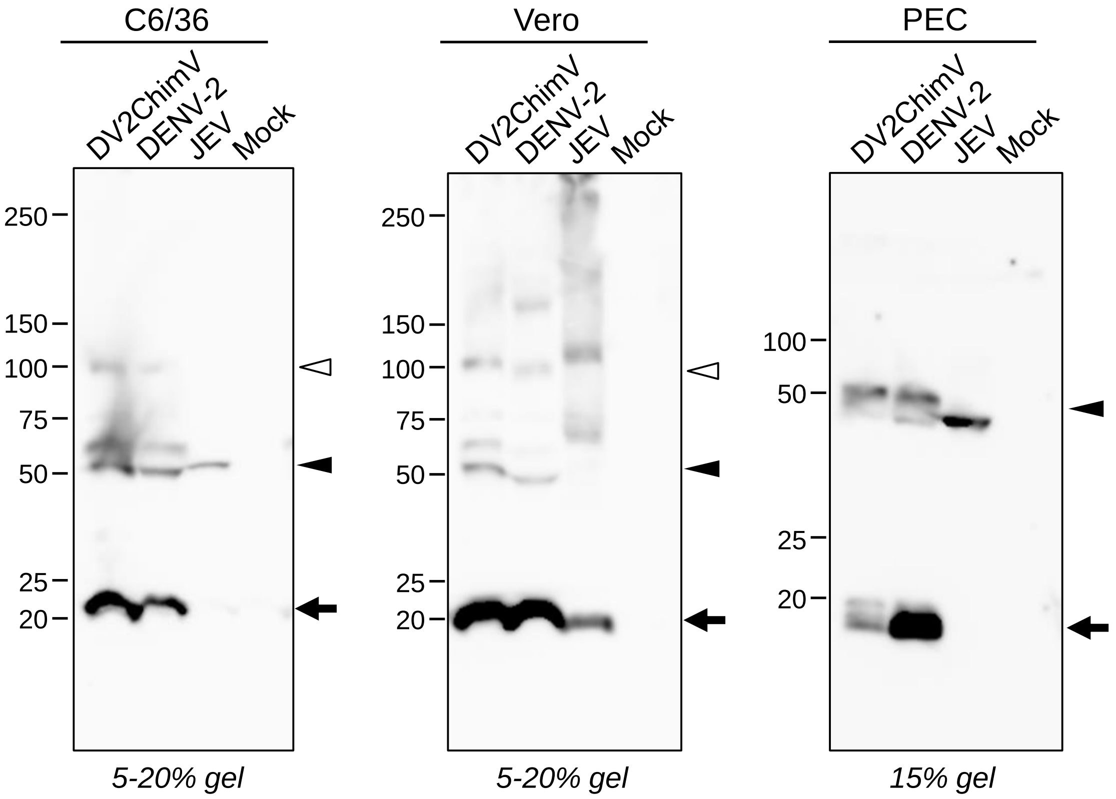

Supplementary Figure S4.
Western blot with anti-E(top) and anti-prM (bottom) antibodies using DV2ChimV, DENV-2, and JEV derived from C6/36 cells, Vero cells, and PEC derived from IFN-α/βR–γR dKO mice. Culture supernatant containing an equivalent number of FFU (5 × 10^5 ffu) was precipitated by methanol/chloroform. For mock, a precipitate was produced from an equivalent volume of culture medium containing 2% FCS. Subsequently, 2 × dodecyl sulfate buffer (ME-)(FUJIFILM Wako Pure Chemical Corporation, Osaka, Japan) was added, and samples were heated to 100 ° C for 10 min,

and loaded onto 12.5% e-PAGEL (ATOO, Tokyo, Japan). After electrophoresis, protein was transferred from the gel to Immun-Blot PVDF membrane (Bio-rad, Hercules, CA, USA). Membranes were incubated withblocking buffer (TBST buffer containing 5% skim milk), and stained with anti-E (D23-1G7C2)(0.1μg/mL) and anti-prM (D25-4D4F10)(0.1μg/mL) (references 17 and 19). Following three rinses in TBST buffer, membranes were incubated with horseradish peroxidase-conjugated anti-Human IgG (Jackson ImmunoResearch, West Grove, PA, USA)(×10,000 dilution). After three additional rinses, the blots were developed with SuperSignal West Dura Extended Duration Substrate (Thermo Fisher Scientific, Waltham, MA, USA), and analyzed by LAS-3000 Imaging System (FUJIFILM). The arrows indicate uncleaved prM proteins. The filled and open arrowheads indicate E proteins and dimerized E proteins, respectively. Data represent a representative experiment from three independent experiments.
